# A possible role for leukotriene B4 in head and neck cancer.

**DOI:** 10.1038/bjc.1989.172

**Published:** 1989-05

**Authors:** I. el-Hakim, J. Zakrzewski, J. Langdon, P. Piper, J. Costello


*Br. J. Cancer* (1989), **59**, 833 © The Macmillan Press Ltd., 1989

LETTER TO THE EDITOR

### A possible role for leukotriene $B_4$ in head and neck cancer

**Sir** – Leukotriene $B_4$ ($LTB_4$) formed through 5-lipoxygenation of arachidonic acid shows potent biological actions aligned to the high incidence of immunological deficiencies and inflammatory symptoms associated with human head and neck cancer (Papenhausen *et al.*, 1979; Bray, 1983). Although homogenates of human squamous cell carcinomas (HSCC) contain 5, 12 and 15 hydroxy-eicosatetraenoic acids the presence of LTS has yet to be established (El-Attar *et al.*, 1985).

Tumour tissue was obtained from the cheek pouch of nine Syrian hamsters after 12 weeks of three applications of dimethylbenzanthracene (DMBA 0.5% in mineral oil) per week as previously described (Eveson, 1981). Animals painted with vehicle alone served as controls. Human studies were carried out in cancerous and unaffected control tissue from eight patients undergoing resection for squamous cell carcinoma of the head and neck. Clinical indices were routinely measured and previous treatments recorded.

Each tissue was extracted in ice-cold ethanol (80%) in the presence of $^3H$-$LTB_4$ (2 nCi, 32 Ci mmol$^{-1}$) for recovery estimation. Samples were then purified by reverse-phase high performance liquid chromatography (Mathews *et al.*, 1981) and $LTB_4$ was measured by specific radioimmunoassay using a double antibody technique (Hayes *et al.*, 1983).

Our results (Table I) show the presence of $LTB_4$-ir in animal and HSCC in amounts similar to that observed for other arachidonic acid metabolites, of which prostaglandin $E_2$ ($PGE_2$) has received much attention (Porteder *et al.*, 1984). A likely source of $LTB_4$ could be inflammatory or malignant cells.

The effects of 5-lipoxygenase products on tumour biology are not well understood but a role for $LTB_4$ as a mediator of inflammation (Ford-Hutchinson *et al.*, 1980), and immunoregulation (Rola-Pleszczynski & Sirios, 1983) has been proposed. In the inflammatory response, $LTB_4$ stimulates increased vascular permeability and oedema responses particularly in the presence of vasodilator substances such as $PGE_2$ (Wedmore & Williams, 1980). $LTB_4$ significantly inhibits human mitogen-induced lymphocyte proliferation *in vitro* probably provided through $PGE_2$ release leading to immunosuppression (Rola-Pleszczynski & Sirios, 1983) and as patients with head and neck cancer show a low level of immune competence this may be important.

**Table I** Measurement of $LTB_4$ immunoreactivity in squamous cell carcinoma of the oral cavity

| | *Hamster* | | *Human* | |
|---|---|---|---|---|
| | *Control* (*n*=*10*) | *Tumour* (*n*=*9*) | *Unaffected tissue* (*n*=*8*) | *Tumour* |
| Tissue weight (g) | 0.06±0.04 | 0.5±0.07 | 0.04±0.14 | 0.04±0.08 |
| $LTB_4$-ir (ng g$^{-1}$) | 0.05±0.3 | 14.7±4.0[a] | 1.5±1.0 | 17.6±5.9[b] |
| Recovery (%) | 52.8±6.0 | 43.0±3.0 | 53.3±7.1 | 46.0±5.8 |

[a] $P<0.01$ compared with control values (Wilcoxon unpaired rank sum test).
[b] $P<0.05$ compared with unaffected tissue (Wilcoxon paired rank sum test).

The observation that LTs can protect cancerous tissue against radiation therapy could have pathogenic and clinical implications (Hansen, 1987). If $LTB_4$ does play a role in the pathogenesis of head and neck cancer, the effects of pharmacological modification of its actions should be evaluated. Yours etc.,

I. El-Hakim[1], J. Zakrzewski[2], J. Langdon[1], P. Piper[3] & J. Costello[2]
[1]Department of Oral and Maxillofacial Surgery, King's College School of Medicine and Dentistry, University of London, London SE5, UK;
[2]Department of Thoracic Medicine, King's College School of Medicine and Dentistry, University of London, UK; and
[3]Department of Pharmacology, Royal College of Surgeons of England, UK.

This study is supported by a grant from the Egyptian government.

Correspondence: Dr I. El-Hakim.

### References

BRAY, M.A. (1983). The pharmacology and pathophysiology of leukotriene $B_4$. *Br. Med. Bull.*, **39**, 249.

EL-ATTAR, T.M., LIN, H.S. & VANDERHOCK, J.G. (1985). Biosynthesis of prostaglandins and hydroxy fatty acids in primary squamous carcinoma of head and neck. *Cancer*, **27**, 255.

EVESON, J.W. (1981). Animal models of intra-oral chemical carcinogenesis. *J. Oral Pathol.*, **10**, 129.

FORD-HUTCHINSON, A.W., BRAY, M.A., DOIG, M.V. and 2 others (1980). Leukotriene $B_4$, a potent chemokinetic and aggregating substance released from polymorphonuclear leukocytes. *Nature*, **286**, 264.

HANSEN, W.R. (1987). In *Prostaglandins and Lipid Metabolism in Radiation Injury*, Hughes, H.N. & Walden, T.C. (eds) p. 233. Plenum Press: New York and London.

HAYES, E.C., LOMBARDO, D.L., GIRARD, Y. and 6 others (1983). Measuring leukotrienes of slow reacting substance of anophyloxis: development of a specific radioimmunoassay. *J. Immunol.*, **131**, 429.

MATHEWS, W.R., ROKACH, J. & MURPHY, R.C. (1981). Analysis of leukotrienes by high-pressure liquid chromatography. *Anal. Biochem.*, **118**, 96.

PAPENHAUSEN, P.R., KUKAWA, A. & CROFT, C.B. (1979). Cellular immunity in patients with epidermoid cancer of the head and neck. *Laryngoscope*, **89**, 538.

PORTEDER, H., MATEJKA, M., ULRICH, W. & SINZINGER, H. (1984). The cyclo-oxygenase and lipoxygenase pathways in human oral cancer tissue. *J. Max.-Fac. Surg.*, **12**, 145.

ROLA-PLESZCZYNSKI & SIRIOS, P. (1983). In *Leukotrienes and Other Lipoxygenase Products*, Piper, P.J. (ed.) p. 234. Research Studies Press.

WEDMORE, C.V. & WILLIAMS, T.J. (1980). Control of vascular permeability by polymorphonuclear leukocytes in inflammation. *Nature*, **289**, 646.


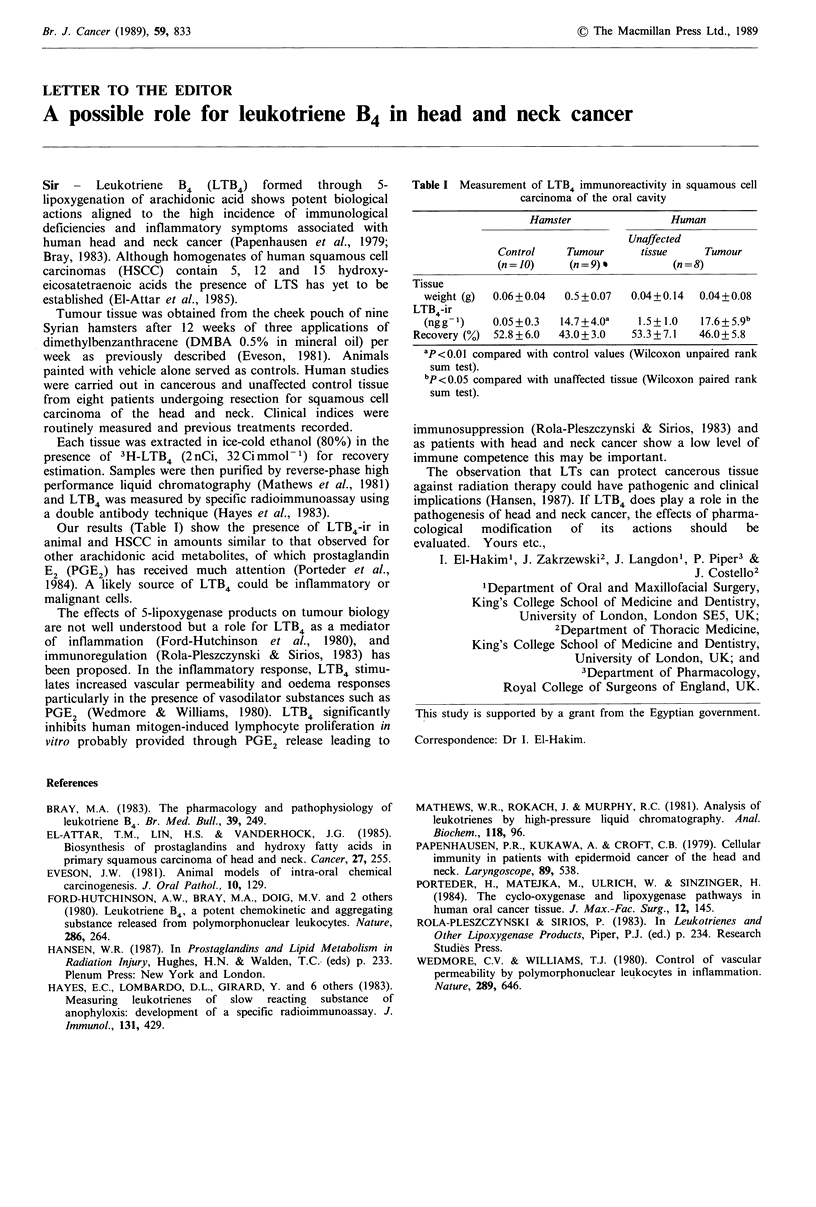


## References

[OCR_00109] Bray M. A. (1983). The pharmacology and pathophysiology of leukotriene B4.. Br Med Bull.

[OCR_00117] Eveson J. W. (1981). Animal models of intra-oral chemical carcinogenesis: a review.. J Oral Pathol.

[OCR_00121] Ford-Hutchinson A. W., Bray M. A., Doig M. V., Shipley M. E., Smith M. J. (1980). Leukotriene B, a potent chemokinetic and aggregating substance released from polymorphonuclear leukocytes.. Nature.

[OCR_00132] Hayes E. C., Lombardo D. L., Girard Y., Maycock A. L., Rokach J., Rosenthal A. S., Young R. N., Egan R. W., Zweerink H. J. (1983). Measuring leukotrienes of slow reacting substance of anaphylaxis: development of a specific radioimmunoassay.. J Immunol.

[OCR_00138] Mathews W. R., Rokach J., Murphy R. C. (1981). Analysis of leukotrienes by high-pressure liquid chromatography.. Anal Biochem.

[OCR_00143] Papenhausen P. R., Kukwa A., Croft C. B., Borowiecki B., Silver C., Emeson E. E. (1979). Cellular immunity in patients with epidermoid cancer of the head and neck.. Laryngoscope.

[OCR_00148] Porteder H., Matejka M., Ulrich W., Sinzinger H. (1984). The cyclo-oxygenase and lipoxygenase pathways in human oral cancer tissue.. J Maxillofac Surg.

[OCR_00158] Wedmore C. V., Williams T. J. (1981). Control of vascular permeability by polymorphonuclear leukocytes in inflammation.. Nature.

[OCR_00113] el Attar T. M., Lin H. S., Vanderhoek J. Y. (1985). Biosynthesis of prostaglandins and hydroxy fatty acids in primary squamous carcinomas of head and neck in humans.. Cancer Lett.

