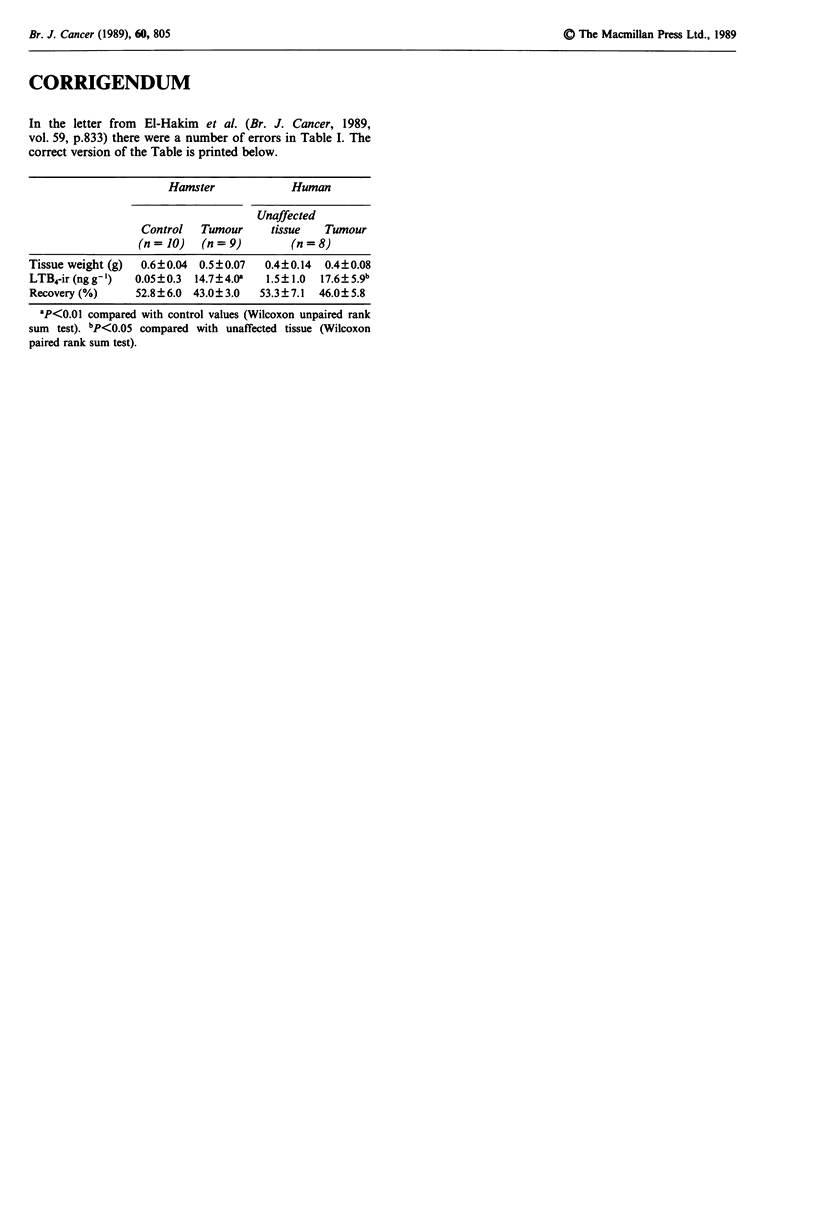# Corrigendum

**Published:** 1989-11

**Authors:** 


*Br. J. Cancer* (1989), **60**, 805 © The Macmillan Press Ltd., 1989

### CORRIGENDUM

In the letter from El-Hakim *et al.* (*Br. J. Cancer*, 1989, vol. 59, p.833) there were a number of errors in Table I. The correct version of the Table is printed below.

| | *Hamster* | | *Human* | |
|---|---|---|---|---|
| | *Control (n = 10)* | *Tumour (n = 9)* | *Unaffected tissue* | *Tumour* |
| | | | *(n = 8)* | |
| Tissue weight (g) | 0.6±0.04 | 0.5±0.07 | 0.4±0.14 | 0.4±0.08 |
| $LTB_4$-ir (ng $g^{-1}$) | 0.05±0.3 | 14.7±4.0[a] | 1.5±1.0 | 17.6±5.9[b] |
| Recovery (%) | 52.8±6.0 | 43.0±3.0 | 53.3±7.1 | 46.0±5.8 |

[a]$P$<0.01 compared with control values (Wilcoxon unpaired rank sum test). [b]$P$<0.05 compared with unaffected tissue (Wilcoxon paired rank sum test).